# Real-time polarization microscopy of fibrillar collagen in histopathology

**DOI:** 10.1038/s41598-021-98600-w

**Published:** 2021-09-24

**Authors:** Adib Keikhosravi, Michael Shribak, Matthew W. Conklin, Yuming Liu, Bin Li, Agnes Loeffler, Richard M. Levenson, Kevin W. Eliceiri

**Affiliations:** 1 Laboratory for Optical and Computational Instrumentation, Department of Biomedical Engineering, University of Wisconsin-Madison, Madison, WI 53706 USA; 2 Marine Biological Laboratory, University of Chicago, Woods Hole, MA 02543 USA; 3 Deparment of Cell and Regenerative Biology, University of Wisconsin-Madison, Madison, WI 53706 USA; 4 Morgridge Institute for Research, Madison, WI 53715 USA; 5 Department of Pathology, MetroHealth Medical Center, Cleveland, OH 44109 USA; 6 Department of Pathology and Laboratory Medicine, UC Davis Health, Sacramento, CA 95817 USA; 7 Department of Medical Physics, University of Wisconsin-Madison, Madison, WI 53705 USA

**Keywords:** Optical imaging, Cancer imaging

## Abstract

Over the past two decades, fibrillar collagen reorganization parameters such as the amount of collagen deposition, fiber angle and alignment have been widely explored in numerous studies. These parameters are now widely accepted as stromal biomarkers and linked to disease progression and survival time in several cancer types. Despite all these advances, there has not been a significant effort to make it possible for clinicians to explore these biomarkers without adding steps to the clinical workflow or by requiring high-cost imaging systems. In this paper, we evaluate previously described polychromatic polarization microscope (PPM) to visualize collagen fibers with an optically generated color representation of fiber orientation and alignment when inspecting the sample by a regular microscope with minor modifications. This system does not require stained slides, but is compatible with histological stains such as H&E. Consequently, it can be easily accommodated as part of regular pathology review of tissue slides, while providing clinically useful insight into stromal composition.

## Introduction

Collagen, the most abundant protein in vertebrates, forms the structural network of the extracellular matrix (ECM) in biological tissues. Structural organization and distribution of the fibrillar types of the collagen family are important factors underlying the properties of tissues and play an integral role in many diseases including cancer. Collagen organization has been shown to be a promising biomarker for evaluation of wound healing, kidney cancer, ovarian cancer, aging, and other processes including atherosclerosis and diabetes^1–7^. Tumor Associated Collagen Signatures (TACS) were first described as alterations in collagen orientation and deposition during mouse mammary tumor progression^8,9^. TACS-3, a pattern describing highly aligned collagen fibers perpendicular to tumor boundaries, was found to be negatively prognostic in human breast cancer^1^. Similar structural signatures were found in other cancer types such as skin^10,11^, ovarian^12,13^, prostate^14^, and pancreas^15,16^.

A variety of established methods currently exist to visualize collagen fibers in different tissue types. Many of these methods can be used in thin-sectioned histological samples, including: (1) absorbance-based stains, e.g., Movat’s pentachrome and Masson’s trichrome, as well as immunohistochemistry^17–21^; (2) polarization-based microscopy, either of native collagen without^22–26^ or with picrosirius red (PSR) staining^27,28^, or more recently described birefringence-sensitive approaches, including LC-PolScope^24^ and polychromatic polarization microscope^25^. Other techniques for visualizing collagen, such as electron microscopy^31^, atomic force microscopy^32^, and scanning electron microscopy^33^ have largely been limited to use in specialized research groups.

Second harmonic generation imaging (SHG)^34–36^ is considered to provide collagen-distribution ground truth. The non-centrosymmetric structure of fibrillar collagen, which is necessary for producing second harmonic signal, makes SHG highly specific to this molecule^35^. Because of its specificity, resolution, label-free detection, and ability to optically section, SHG has become a popular fibrillar collagen imaging method. However, high cost, complexity, and the need for experienced optics experts make it impractical for use in clinical (pathological) assessment of collagen orientation in resected cancer tissues. Undoubtedly, SHG has great advantages in terms of imaging depth and higher-order information using forward-to-backward intensity ratios^37^ and polarization-based studies^13,38,39^. However, given the financial and temporal constraints on pathology practice, where even the performance of histochemical stains such as trichrome or picrosirius red introduce delays in generating clinically relevant assessments of patient tissue, the advantages mentioned above are not sufficient to drive usage in routine patient care^1,7,15,16,40,41^.

In previous studies, we have shown that LC-PolScope^42,43^, a highly sensitive polarization imaging system, can be used for imaging collagen in histopathology slides without the need for intensifying birefringence signals through picrosirius red staining. This polarization tool yields comparable results to SHG when quantifying fiber orientation or alignment^29,44^. Although simpler and very cost effective compared to SHG imaging and also label free, this modality still requires modifications to the pathologist’s microscope by the addition of a chromatic filter, special variable retarders, and circular and linear polarizers. Most importantly, the final image is rendered computationally with specialized software that adds steps and makes it more difficult for incorporation into clinical practice. Clinical histopathology relies on observation of disease features by trained pathologists using conventional microscopes, with minimal manipulation of the tissue after it is stained with H&E. Hence, to maximize adoption there is a need to find the most accessible collagen visualization method that can be incorporated in everyday pathology routine.

Despite all the advancements in imaging fibrillar collagen at different scales and based on different molecular and optical properties, and despite the proven importance of collagen in disease—including cancer—development and progression, assessment of collagen remains underutilized in clinical diagnosis. To address this need and to provide a real-time method that can demonstrate collagen through the eyepieces of a pathologist’s microscope, we introduce a novel use of polychromatic polarization microscope (PPM)^30,45^. PPM is a polarization microscopy method previously introduced for high-resolution imaging of retardance and optical axis orientation in birefringent samples. This method is a hardware-based polarization solution that can be added to conventional histopathology microscopes and does not require the use of any specialized software. PPM, once installed, can be used with the ease of polarizers already familiar to most histopathologists. Except for the necessary polychromatic optics, no other specialized hardware is required and a straightforward workflow is involved for collecting birefringent images. Here we first demonstrate that quantification of fibrillar collagen images acquired from PPM and SHG imaging yield statistically similar results. More importantly, we also show that PPM is capable of visualizing fibrillar collagen, colored by fiber orientation, while inspecting an H&E-stained tissue sample through the eyepiece by clinicians. This is the only imaging approach we know that allows a pathologist to specifically visualize collagen in real time while inspecting H&E features. We demonstrate by comparing to gold standard methods and quantitation that this approach allows for the clear visualization of fibrillar collagen topology while also presenting classic H&E histology.

## Results

For our primary data we use images from two TMAs containing breast and pancreas specimens that have been examined by our group in previous studies and been shown to contain quantifiable fibrillar collagen-based prognostic markers. In addition to these datasets, additional clinical histopathology samples were examined; it was possible to visualize collagen in those as well. While these samples were not previously investigated for fibrillar collagen, they help demonstrate the ubiquity of collagen in histology and the broad potential utility for this method.

### Quantitative comparison between PPM and SHG imaging of collagen

**Figure 1:**
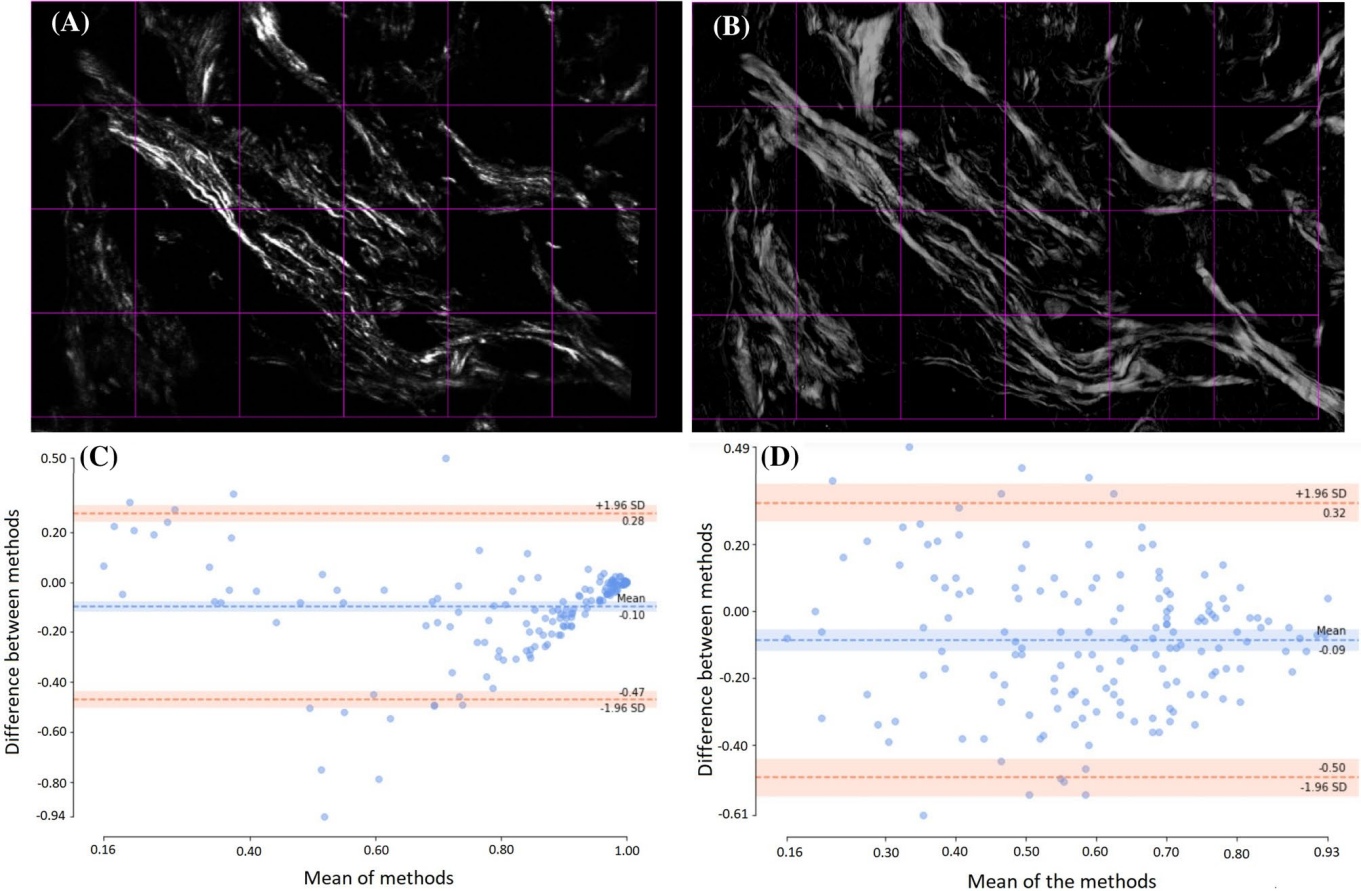
Top row shows the ROI selection for fiber metrics analysis based on 10% SHG signal for SHG (**A**) and monochromatic differential PPM (**B**) images. Bottom row shows the Bland–Altman analysis plot for sin() of orientation of the fibers inside blocks as the difference of two measurement vs. the mean (**C**) and the same plot for fiber alignment inside ROI (**D**). Each block is 256 × 256 pixels and total size of the images is 186 × 280 µm**.**

Collagen fiber orientation and alignment have been extensively explored and accepted as biomarkers for disease progression and patient prognosis via mechanisms connecting alteration in tumor cell signaling with the surrounding microenvironment^1,15,36,41,46^. So, it is important to verify that collagen images acquired using PPM and SHG imaging can statistically yield the same result when we quantify fiber orientation and alignment. SHG images were divided into blocks of 256 × 256 pixels and those with less than 10% second harmonic signal were excluded from analysis. An example of selected ROIs is illustrated in Fig. 1A,B that show SHG and monochromatic differential PPM images of the same ROI, respectively. This resulted in 159 image blocks (each 256 × 256 pixels) for both SHG and monochromatic differential PPM images (described in “Methods” and Fig. 7). Images were analyzed using our widely used, lab-developed fiber analysis software CurveAlign^47,48^ to calculate fiber orientation and fiber alignment for each block^47^. Fiber orientation was defined as the angle with respect to the horizontal axis, which causes angle ambiguity between the angles around the lower and upper limits. More specifically, angles close to 0° and 180° essentially indicate similar orientations, but the absolute angle differences are significant. Hence a sinusoid function $\mathrm{sin}\left( x\right)$ (x is the orientation value in degree) was used to map the orientation values from [0 180] degrees to [0, 1] to avoid ambiguity. Collagen alignment coefficient ranging from 0.0 to 1.0 indicates how similarly the orientations of collagen fibers are distributed in a given area. It is defined as the mean resultant vector length in circular statistics^49^, with 1.0 indicating all fibers are aligned in one direction, while small values close to 0.0 indicate fibers are oriented in random directions.

A Bland–Altman plot showing the difference between two measurements of the same parameter vs. the average of these values is often used to describe the agreement between two quantitative measurements by constructing limits of agreement^50,51^. Bland–Altman plots of sinusoids of orientation and alignment for both image sets are shown in Fig. 1C,D. Blue dotted lines show the mean of differences or constant bias between two measurements and the red lines show the 95% confidence interval (CI) (mean ± 1.96 STD). The bias between our measurements is negligible, which suggests no systematic difference between measurements from collagen fibers as seen by SHG or PPM microscopy. For both orientation and alignment, differences between two measurements of more than 95% of the data fall between ± 1.96 STD, which is proposed as a good agreement between two methods by Bland and Altman^50^.

### Visualizing collagen reorganization in breast cancer

The polychromatic polarization microscope can readily visualize collagen fibers using clinical microscopes and during pathological assessment of the samples for diagnosis and prognosis. This will allow pathologists to incorporate a qualitative measure of stromal biomarkers, such as fiber deposition^7,16,52,53^, orientation and alignment^1,7,15,36,41^. All other currently described approaches for visualizing collagen require disruption to the clinical pathologist workflow.

**Figure 2:**
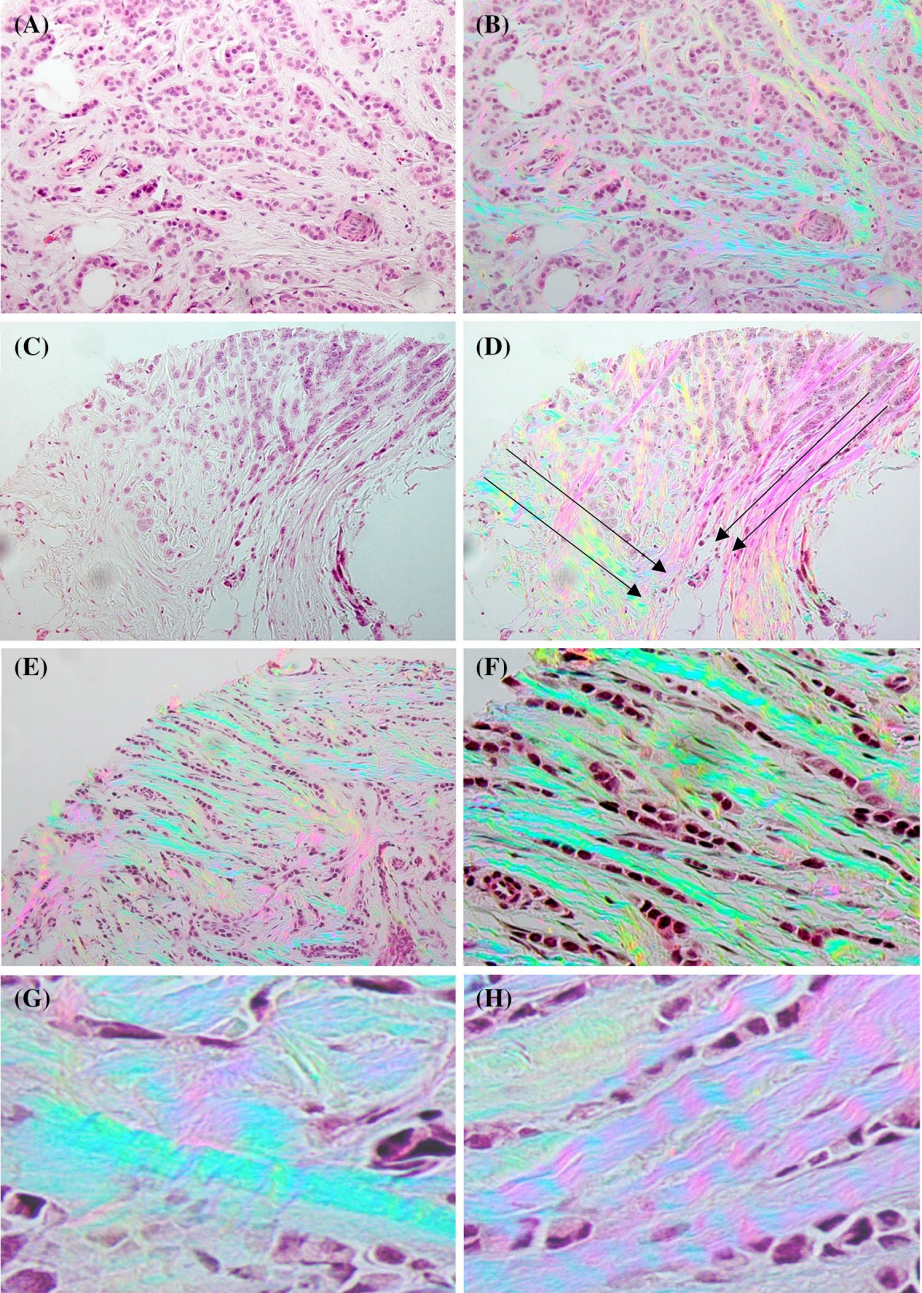
Collagen compartmentalizes the group of tumor cells that looks like a big mass of cells if one only looks at H&E images (**A**, **C**). (**D**) Shows how the cell migration direction in an invasive ductal adenocarcinoma (IDC) can be visualized using PPM while this information cannot be readily extracted by just looking at BF image of H&E stained tissue (**C**). (**E**) and (**F**) Show that the difference in fiber thickness doesn’t change the color and color is only related to the fiber orientation (all blue fibers with different thickness) diameters flowing from top left corner to the bottom right corner are depicted as green. (**G**) and (**H**) Show curvy fibers result in periodic changes and this can be used as a comparison between straight and curvy fibers. The tissue area represented in images (**A**) to (**E**) is 1384 × 1038 µm, the area in image (**F**) is 692 × 519 µm and in images (**G**) and (**H**) is 346 × 259 µm. Images shown are purposely not post-processed in order to represent what the pathologist would see looking through the eyepiece.

Figure 2 shows how PPM can provide real-time visualization of the presence of fibrillar collagen in two human breast samples diagnosed with invasive ductal adenocarcinoma (IDC). Collagen compartmentalization of the tumor cells, flow direction of tumor cells along collagen produced pathways besides fibrillar collagen orientation and alignment can be easily seen by pathologist while examining the tissue sample. However, regular clinical histopathology practice that only utilizes the bright-field image of the tissue, either digitally or through the microscope eyepieces, can miss such ECM-related information.

The colors visualized in traditional polarization microscopy images of picrosirius red-stained samples, depend not only on fiber orientation but also fiber thickness^54^. However, in images acquired by PPM, colors visualized depend only on the orientation of optical axis, which in case of fibrillar collagen is the same as the fiber orientation. In this image all collagen fibers of different diameters are represented as green. PPM is highly sensitive to changes in fiber orientation, which generate the different colors seen. The bottom row shows the color variation along curvy fibers (periodic purple and blue hues) in contradistinction to straight fibers that maintain the same hue (green) along their length.

**Figure 3:**
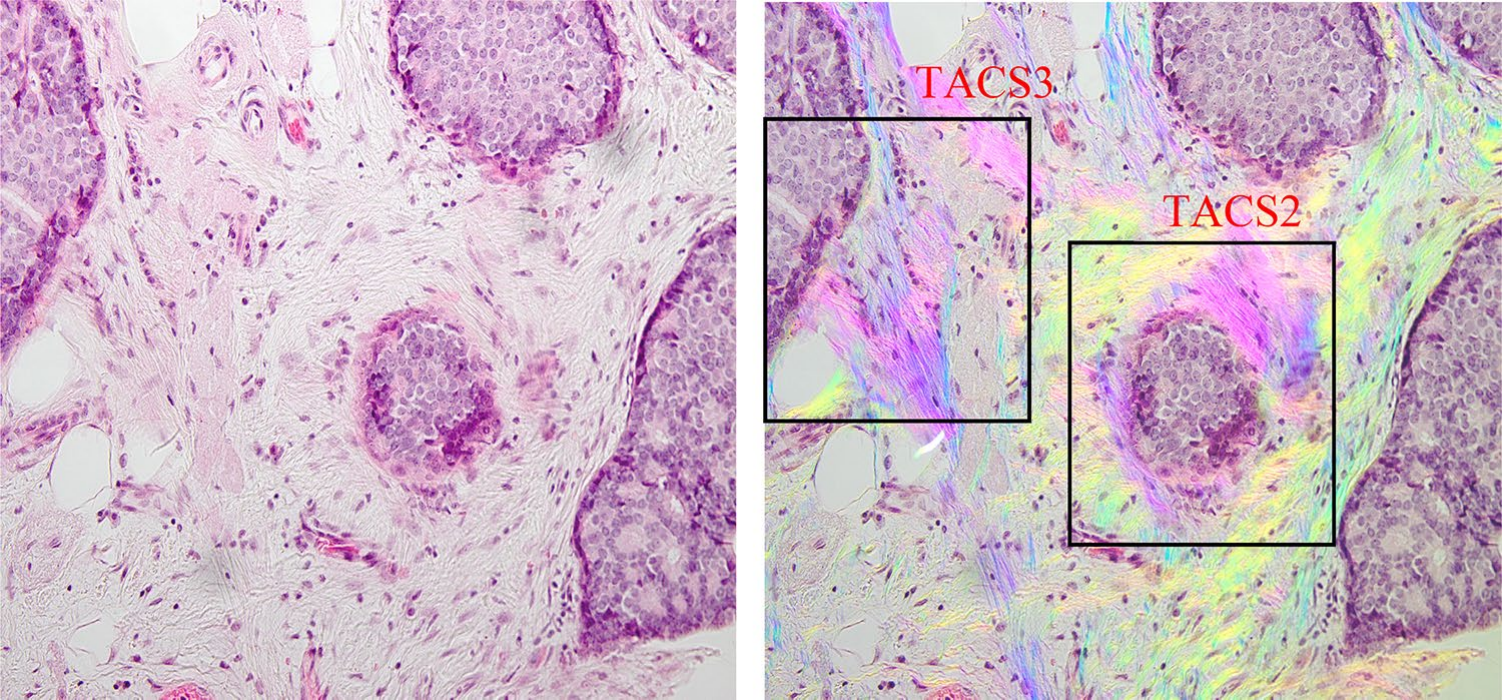
TACS-2 and -3 are both present in this figure. These collagen fibers are easily identifiable in the PPM image (right) by a color contrast compared to other tissue parts, the highly aligned fibers are also seen with similar hue (purple) that are perpendicular to tumor boundary in TACS-3 region and tangential to tumor boundary in TACS-2 regions, while none of these information can be readily extracted from the BF image only. The tissue area represented in images is 692 × 519 µm.

Tumor Associated Collagen Signatures (TACS) have been used to describe different fibrillar collagen patterns near the tumor boundary during mouse mammary cancer progression^8,9^. TACS-1 indicated a finding of long and straight fibers tangential to tumor boundary, while TACS-2 category indicated the presence of dense and shorter fibers randomly oriented around the tumor boundary. TACS-3 described a pattern consisting of fibers with perpendicular orientation in respect to the tumor boundary, with the biological interpretation that such orientation could facilitate tumor cell migration and metastasis. TACS-3 has been proven to be a prognostic biomarker during breast cancer progression^1^. Figure 3 presents a ductal carcinoma in situ (DCIS) surrounded by both TACS-2 and TACS-3 fibers.

### Collagen fiber visualization in other sample types

**Figure 4:**
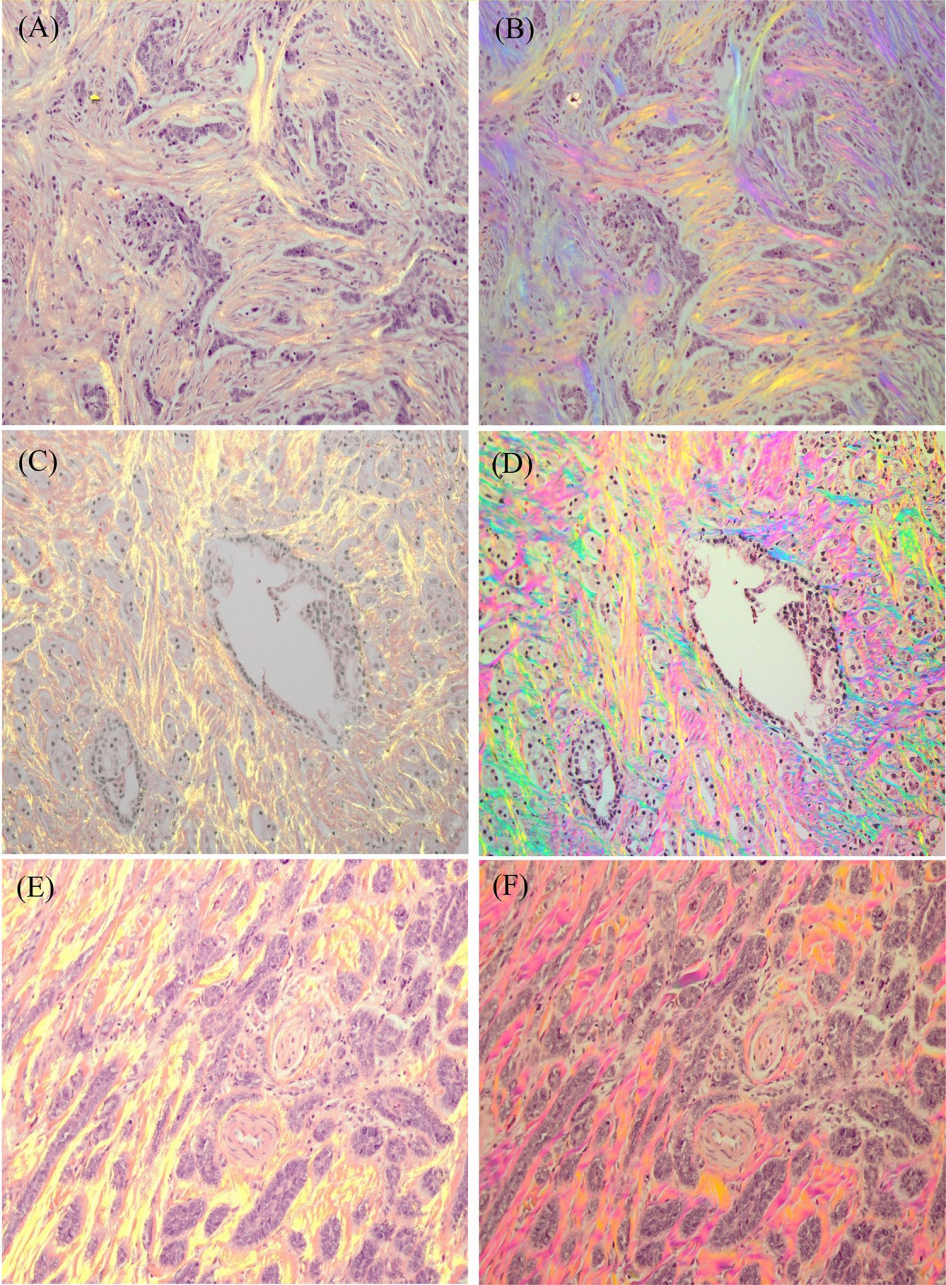
Images in A, C, E show the SHG images in yellow channel overlaid on bright-field images of H&E-stained samples of colon cancer, prostate cancer and basal cell carcinoma, respectively . Images in B, D, F show the same fields of view observed through the eyepiece of a PPM microscope. The third row is a 20× view of a selected ROI from first row, and the tissue area represented in images is 692 × 519 μm.

To further demonstrate the PPM’s capability to visualize collagen fibers in different tissue samples, we examined three tissue types previously investigated for stromal response during cancer progression. Figure 4A,B show comparisons between SHG and PPM views of colon cancer, C and D are prostate cancer sample, and E and F show basal cell carcinoma. Left column images show the SHG images of the H&E-stained tissue sections in a yellow channel overlaid on bright-field images of the same field of view, while the right column shows the PPM images of the same fields of view that would be visible through direct viewing using the eyepiece of the microscope. As can be seen in Fig. 4, PPM is highly sensitive to fibrillar collagen and despite the overlaid images (left column) that are displaying collagen in a single color, the colors of PPM images reveal the alignment angles of fibers in these samples.

Increased deposition and highly aligned fibers have been previously described during progression of colonic dysplasia and cancer^55–57^ that is displayed in Fig. 4A,B.

Figure 4C,D show high grade prostate adenocarcinoma, which has been reported to present a reticular structure^58^ during cancer progression, and at high grades the fibers become more aligned such that the alignment is highly correlated with Gleason score of the sample^59^. Figure 4E,F show a similar representation for a basal cell carcinoma sample that has been attributed to lower collagen content compared to normal skin and replacement of fibers by tumor cells^10,11^.

**Figure 5:**
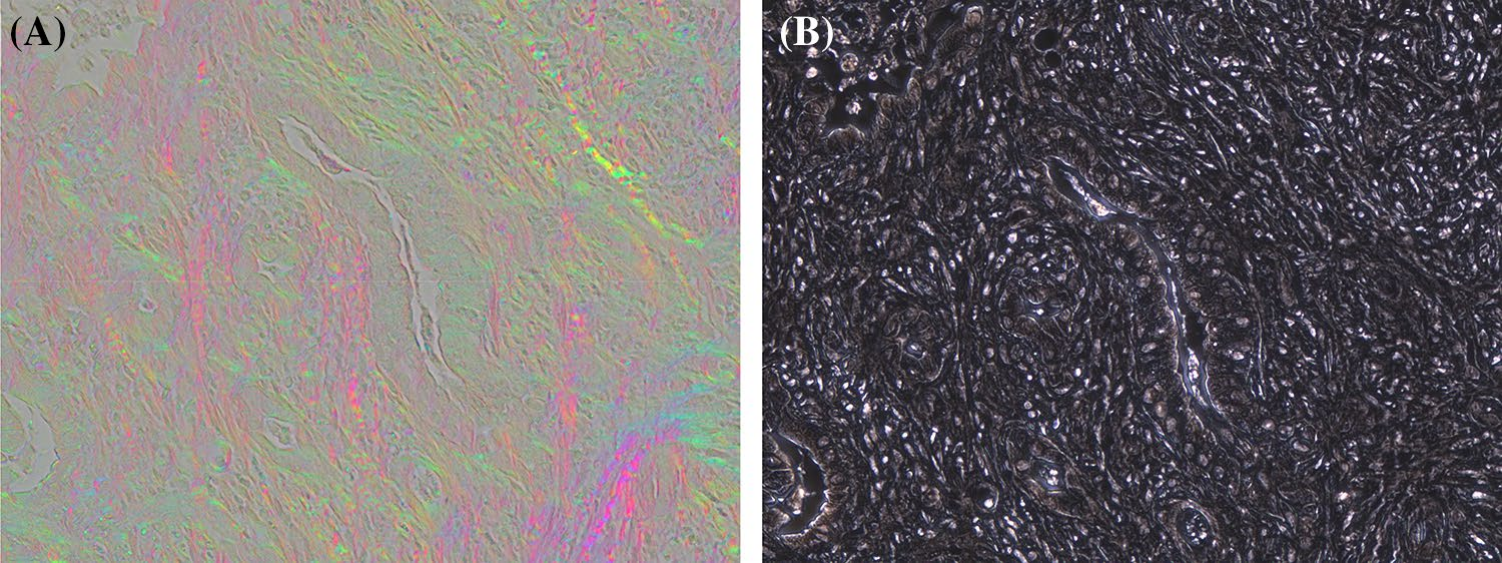
(**A**) Shows a pancreatic ductal adenocarcinoma surrounded by fibrotic tissue that has been visualized using PPM. (**B**) Shows the phase contrast imaging of the same region to demonstrate the cellular structures. The tissue area represented in images is 433 × 330 µm.

Collagen visualization using PPM is not dependent on staining of the tissue; and despite traditional polarization based imaging that requires picrosirius staining for birefringent enhancement, PPM is capable of visualizing collagen in its native state and in unstained tissue sections. Figure 5A shows a pancreatic ductal adenocarcinoma surrounded by fibrotic tissue that has been visualized using our PPM system. PPM can complement other modalities such as phase contrast imaging (Fig. 5B) that are more sensitive to cellular and nuclear structures to provide a comprehensive map of cellular and extracellular structures, which can be beneficial to both research and surgical pathology applications.

## Discussion

This study presents a novel approach for visualizing fibrillar collagen in histopathological samples using polychromatic polarization microscopy technique. Traditional polarization-based imaging techniques require additional sample preparations such as staining with picrosirius red^28,60^. In a more recent and high-resolution system called LC-PolScope^29,43,44^ the collagen signals are rendered computationally, a step that complicates integration in clinical pathology. Fereidouni et al.^61^ used dual-mode imaging of standard H&E-stained histology samples, combining brightfield and fluorescence imaging to highlight collagen in bright-field images. However, due to need of fluorescence imaging and the computational approach employed, this method can’t be integrated into every pathologists workflow without disrupting current practice. To remove the need of the optical system we have recently developed a convolutional neural network-based method that can generate virtual SHG images directly from bright-field images of H&E-stained slides after training with breast and pancreatic tissue sections ^62,63^, but such models must be verified before use on tissue types outside the training pool. However, PPM can visualize and characterize collagen fibers in H&E-stained slides, aided by the optically generated color signals, and thus provides a unique opportunity for incorporating stromal biomarkers in diagnosis and prognosis.

In research settings, SHG imaging is usually the method of choice for fibrillar collagen imaging, due to its specificity to non-centro-symmetricity, a characteristic of many molecular species of collagen^38^. However, SHG is costly, requires expertise to run effectively and its capacity for imaging at depth is not needed for the thin tissues typically used in histopathology. A simpler implementation of SHG for potential clinical applications has recently been commercialized^64^; nevertheless, its cost (in the hundreds of thousands of dollars) places it out of reach for most centers. Bland–Altman analysis of SHG vs. PPM results indicated that when diagnostic and prognostic collagen biomarkers such as fibrillar orientation and alignment^1,15,51^ are of concern, the two methods yield similar results.

Quantitative analysis of PPM images require the procedures mentioned in the “Methods” section to obtain a differential image, which adds extra steps to the pathological review of the tissue. The real advantage of PPM, however, is to provide underlying stromal information such as collagen abundance, orientation and alignment assisted with optical color rendition of fibers in real time through the eyepieces, available for immediate viewing and interpretation by the pathologist. Fibers represented by similar hues are aligned in a similar direction, and a complete color spectrum, perceptible with human color vision, captures 180 degrees of orientation. Several examples of collagen remodeling were shown here to illustrate the ability of PPM to generate collagen signals useful for lesion categorization. Breast, colon, prostate and skin samples were used to compare fiber visualization using SHG and PPM. Besides the low cost of PPM microscopy vs. SHG systems, its acquisition speed can be orders of magnitude faster, an essential attribute for visualization of large sample areas.

In this proof of concept study, we set out to demonstrate that PPM can be a viable real-time method for visualizing and quantitating fibrillar collagen changes in histopathology studies. We used SHG-based fiber detection as the gold standard; PPM was able to visualize and quantitate the same fibers and generate statistically similar metrics. However, it is also clear that PPM can identify additional fiber content not seen in SHG (Fig. 1). This could be due to a number of contributing factors including nascent collagen fibers, poorly polymerized collagen or other collagen fiber types. Future studies will need to investigate this.

## Methods

### Imaging systems

#### Polychromatic polarization microscope (PPM) imaging

**Figure 6:**
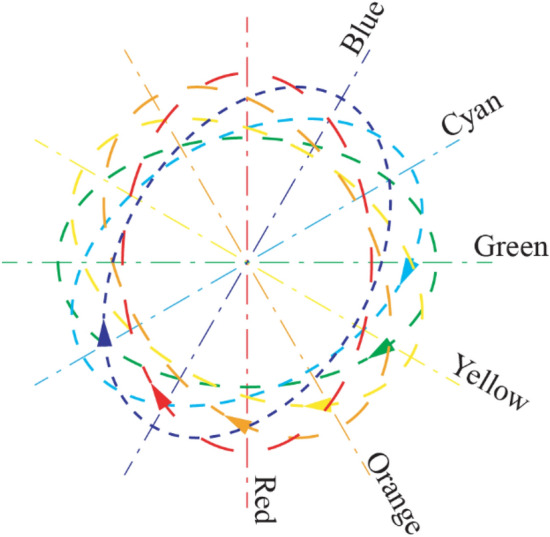
An example of the fan with the right polarization ellipses for visible spectrum.

The PPM is based on a standard microscope with white light illumination, which is equipped with a special polychromatic polarization state generator and achromatic circular analyzer. The polarization state generator produces polarized light with the polarization ellipse orientation determined by the wavelength, which we call the spectral polarization fan. An example of the fan with the right polarization ellipses for the visible spectrum is shown in Fig. 6. All polarization ellipses have the same ellipticity angle e ~ 40°.

If the specimen under investigation isn’t birefringent then the beam passes it without alteration of the polarization. The circular analyzer will evenly, partially transmit all wavelengths, and the output beam will stay white. If the object is birefringent then it modifies the spectral polarization fan. For example, a particle with phase retardation ~ 10° and the fast axis at 45° will add ~ 5° to the red polarization ellipticity angle and subtract ~ 5° from the green ellipticity angle. The red component will have the right circular polarization with ellipticity angle 45°, and it will be extinguished completely by the left circular polarizer. The green component will have an ellipticity angle ~ 40°, and its transmission will be increased in ~ 4 times. So, the birefringent particle will be mostly green. If the particle or the spectral polarization fan is rotated by 90°, the picture becomes complementary and the birefringent particle is mostly red.

**Figure 7:**
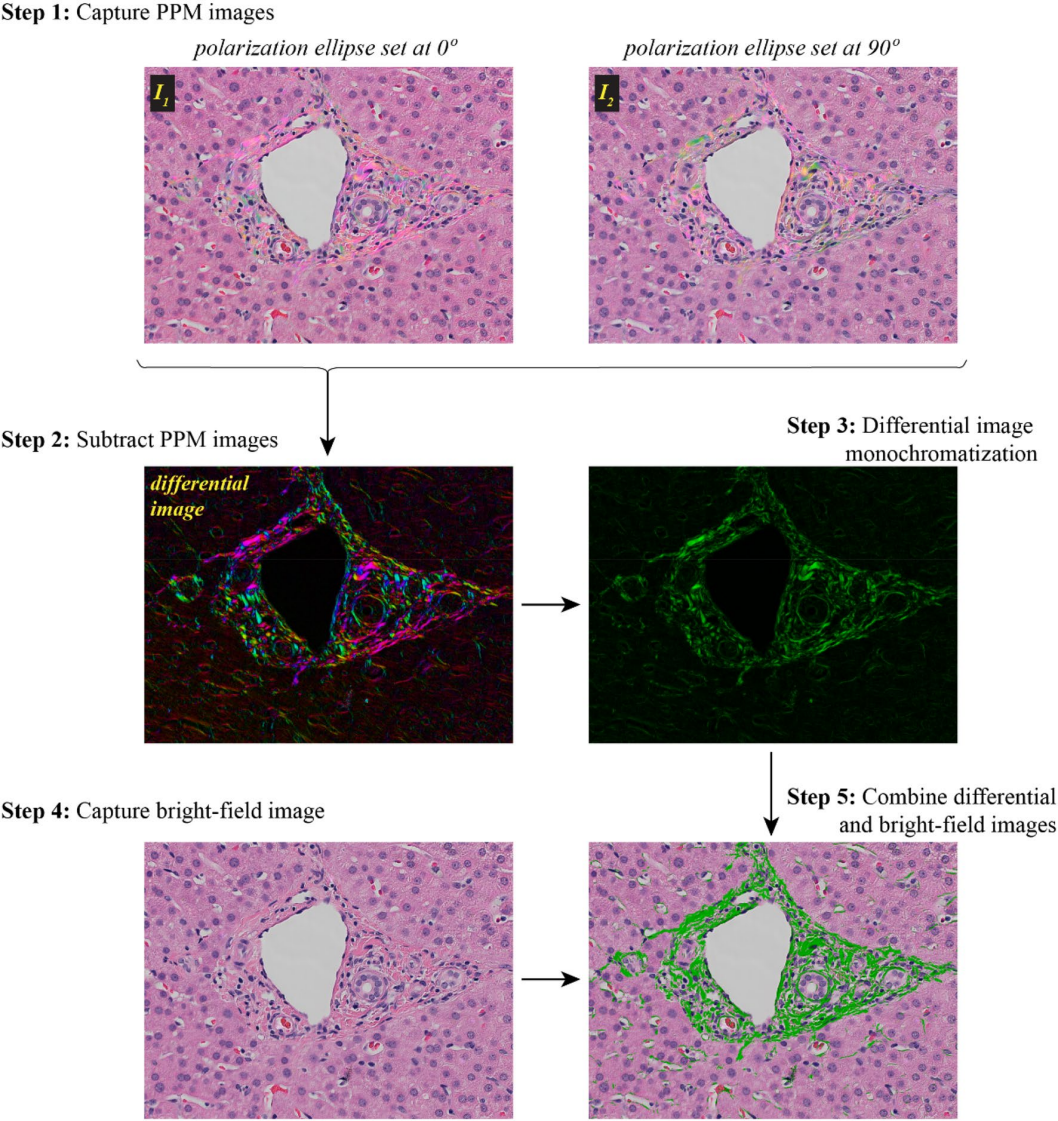
PPM workflow for acquiring birefringence image.

In order to increase the image contrast and suppress the contribution from non-birefringent structures we can subtract one complementary bright-field PPM image from another. A computed differential PPM image depicts colors that are generated by the birefringence only and eliminates the stain colors. For further analysis we can transform the differential PPM image to monochromatic by using its brightness. Then overlap the monochromatic PPM image with conventional brightfield image captured in unpolarized light. This workflow is summarized below, and it is also illustrated in Fig. 7.

**Step 1.** Take two complementary bright-field PPM images with spectral polarization fans at 0° and 90°.

**Step 2.** Compute a difference. The differential PPM image will show the non-birefringent structures in black and the birefringent structures in color.

**Step 3.** Replace the variety of birefringent colors with one monochromatic color, for example by green.

**Step 4.** Take a conventional bright-field image in unpolarized light.

**Step 5.** Combine differential PPM image and conventional bright-field image.

A white balance step can be used to correct the camera hue for these images before computing the result. The image processing code in Python, user instructions, and example data are available at https://github.com/uw-loci/polychromatic-polarization.

For the images in Fig. 7, we used an inverted light microscope Olympus IX81 (Olympus America, Center Valley, PA, USA) equipped with objective lens UPLFL20xP/NA0.5 and 100 W halogen lamp and images were collected using Olympus DP73 color camera. All images were taken in white light without any filter. BF and PPM images in Figs. 4, 5, 6 and 7 were collected using an upright light microscope Olympus BX60 (Olympus America, Center Valley, PA, USA) equipped with objective lens 10x/NA0.3, 20x/NA0.5 and 40x/N0.75 and 100 W halogen lamp and the images were collected using a Olympus DP25 Color CCD camera. Figure 3 was done using data from both systems.

#### Second harmonic generation (SHG) imaging

All the SHG imaging in this study was done with a custom built integrated SHG/bright field imaging system. A MIRA 900 Ti: Sapphire laser (Coherent, Santa Clara, CA) tuned to 780 nm, with a pulse length of less than 200 fs, was directed through a Pockels cell (ConOptics, Danbury, CT, USA), half and quarter waveplates (ThorLabs, Newton, NJ, USA), beam expander (ThorLabs), a 3 mm galvanometer driven mirror pair (Cambridge, Bedford, MA), a scan/tube lens pair (ThorLabs), through a dichroic beam splitter (Semrock, Rochester, NY) and focused by 20X/0.75NA air objective lens (Nikon, Melville, NY). SHG light was collected in the forward direction with a 1.25 NA Abbe condenser (Olympus) and filtered with an interference filter centered at 390 nm with a full width at half maximum bandwidth of 18 nm (ThorLabs MF390-18). The back aperture of the condenser lens was imaged onto the 5 mm aperture of a H7422-40P GaAsP photomultiplier tube (Hamamatsu, Hamamatsu, Japan) the signal from which was amplified with a C7319 integrating amplifier (Hamamatsu) and sampled with an analog to digital converter (Innovative Integration, Simi Valley, CA). Timing between the galvo scanners, signal acquisition, and motorized stage positioning was achieved using our custom software called WiscScan. SHG images were tiled with 5% overlap using automation provided by WiscScan. Stage positions for individual images and pixel size data were read in by Bio-Formats^65^ image metadata and this was then used by the grid/collection stitching ImageJ plugin to reassemble a high-resolution large field of view image of the entire imaged area.

#### Phase contrast microscope

For imaging the unstained pancreatic ductal adenocarcinoma tissue represented in Fig. 5A,B, we used an inverted Olympus microscope IX81. The microscope was equipped with a negative phase contrast objective lens UPLFLN20XPH/NH and Hamamatsu 3 CCD digital color camera C7780-20. The camera sensor has 1344 × 1024 pixels and pixel size 6.45 µm. The total size of the sensitive area is 8.67 mm × 6.60 mm. The corresponding FOV with 20× objective lens is 433 µm × 330 µm. In order to capture a PPM image we took out the phase annulus from the condenser and installed the polychromatic polarization state generator into the microscope (https://www.mbl.edu/bell/files/2014/12/PPM_2017-Shribak.pdf). In order to take a phase image we removed the polychromatic polarization state generator and moved the phase annulus back in the illumination beam.

### Histological samples

For this study we have retrospectively used two well studied tissue microarrays containing breast and pancreatic cancer. Complete description for breast samples can be found in^1^ which demonstrated the prognostic value of the TACS3 in human patient; and for pancreatic samples in^15^, which has shown that collagen alignment is a negatively prognostic factor in pancreatic ductal adenocarcinoma progression. Both breast and pancreatic tissue microarrays were collected and constructed at University of Wisconsin-Madison and all experimental protocols were approved by the University of Wisconsin-Madison institutional research board committee. Informed consent was obtained from all subjects, and if subjects are under 16, informed consent from a parent and/or legal guardian. All methods were carried out in accordance with relevant guidelines and regulations at the University of Wisconsin at Madison.

However, to briefly describe the samples, all tissues were formalin-fixed and paraffin-embedded, then cut into 5 µm thin slices, affixed to a slide and stained with hematoxylin and eosin (H&E) before mounting with a coverslip.
